# Contralateral migration of Kirschner wire from right acromioclavicular joint to left side of neck: a case report

**DOI:** 10.1186/s13256-019-2279-1

**Published:** 2019-12-11

**Authors:** How-Yun Ko, Ka-Wo Lee

**Affiliations:** 1 Department of Otorhinolaryngology – Head and Neck Surgery, Kaohsiung Medical University Hospital, No.100, TzYou 1st Road, Kaohsiung City, 807 Taiwan; 2 Faculty of Medicine, College of Medicine, Kaohsiung Medical University, Kaohsiung, Taiwan; 3 Department of Otorhinolaryngology, Kaohsiung Municipal Ta-Tung Hospital, Kaohsiung, Taiwan

**Keywords:** Kirschner wire, Contralateral migration, Clavicle fracture

## Abstract

**Background:**

Kirschner wire migration is one of the most common complications after internal fixation of fracture or dislocation in the shoulder region. However, cases of contralateral wire migration are rare. We present a case of contralateral loosened Kirschner wire migration from the right acromioclavicular joint to the left side of the neck without damage to any important structures or great vessels.

**Case presentation:**

We report a case of a loosened Kirschner wire migrating from the right acromioclavicular joint to the left side of the neck in a 34-year-old Taiwanese man following a route of transversal, descendant, and then ascendant directions. The Kirschner wire was removed by exploratory neck dissection under C-arm fluoroscopy assistance without complication.

**Conclusion:**

Wire migration may occur after surgical treatment with or without clinical complaint. Several hypotheses for the mechanism of wire migration have been postulated, including muscular activity, respiratory motion, gravity, and motion of upper extremity. Therefore, the importance of follow-up should be communicated to the patient. Once wire loosening or migration is noted, the implant should be removed immediately under intraoperative C-arm fluoroscopy or ultrasound assistance.

## Background

Kirschner wire (K-wire) is one of the most commonly used implants in the management of fracture or dislocation in the shoulder region. Despite good outcomes, there are several complications after K-wire fixation, and wire migration is one of the most frequent complications. Several catastrophic complications had been reported due to wire migration, such as wire penetrations into the heart, spinal cord, lungs, trachea, great vessel, and abdominal cavity [1–6]. Here, we present a patient with contralateral loosened K-wire migration from the right acromioclavicular (AC) joint to the left side of the neck.

## Case presentation

A 34-year-old Taiwanese man presented to our emergency department with a 3-day history of coughing and chest tightness. He had a history of essential hypertension and congestive heart failure followed up at our cardiovascular department and under medication control. He was an express delivery worker with right hand dominance.

**Fig. 1:**
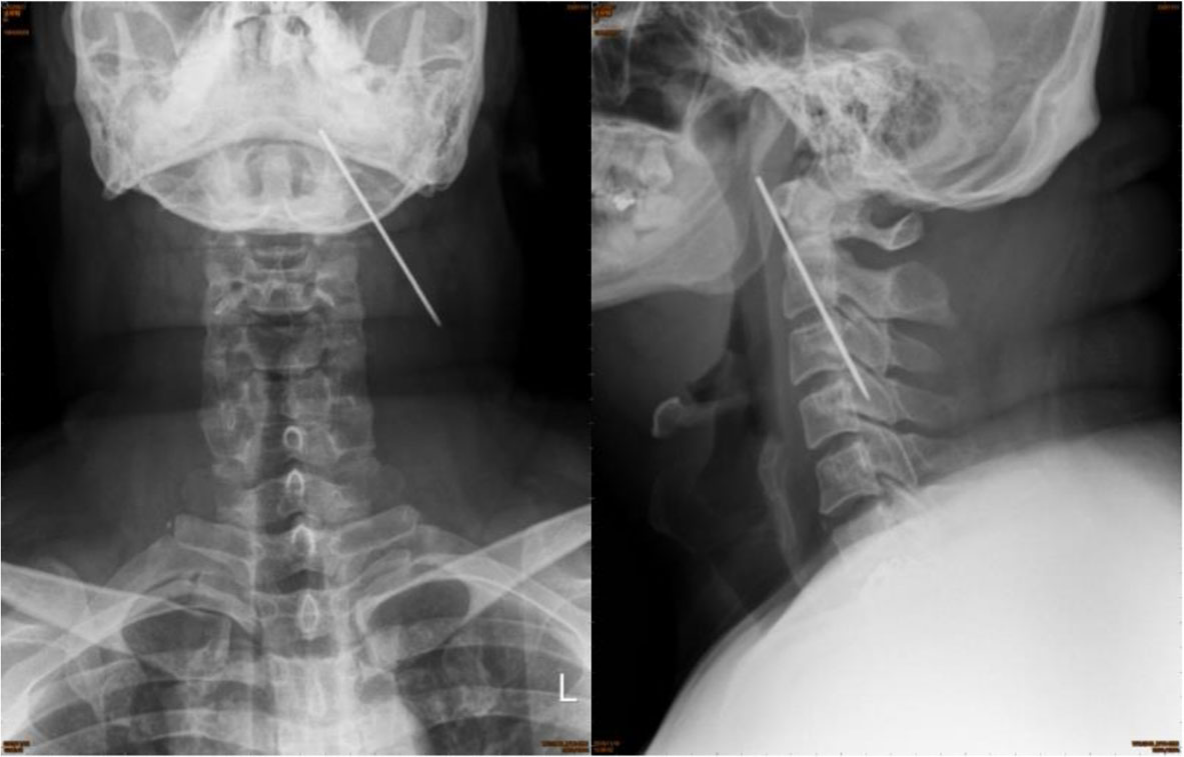
Cervical spine anteroposterior and lateral view revealed foreign body of metallic density crossing over the C2–C4 level on left side of neck

**Fig. 2:**
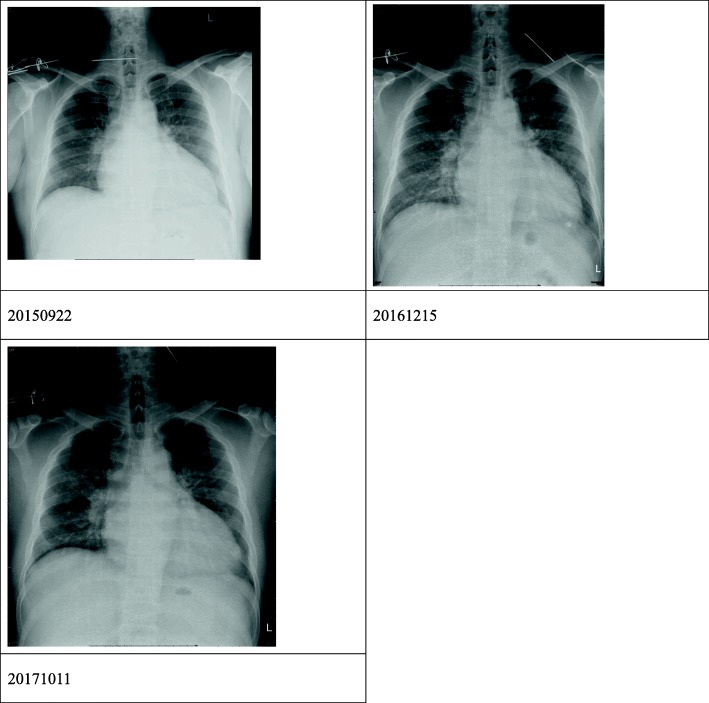
A series of chest-posteroanterior films showing the contralateral migration of the Kirschner wire from right side to the left side of the neck (22 September 2015, 15 December 2016, and 11 October 2017)

**Fig. 3:**
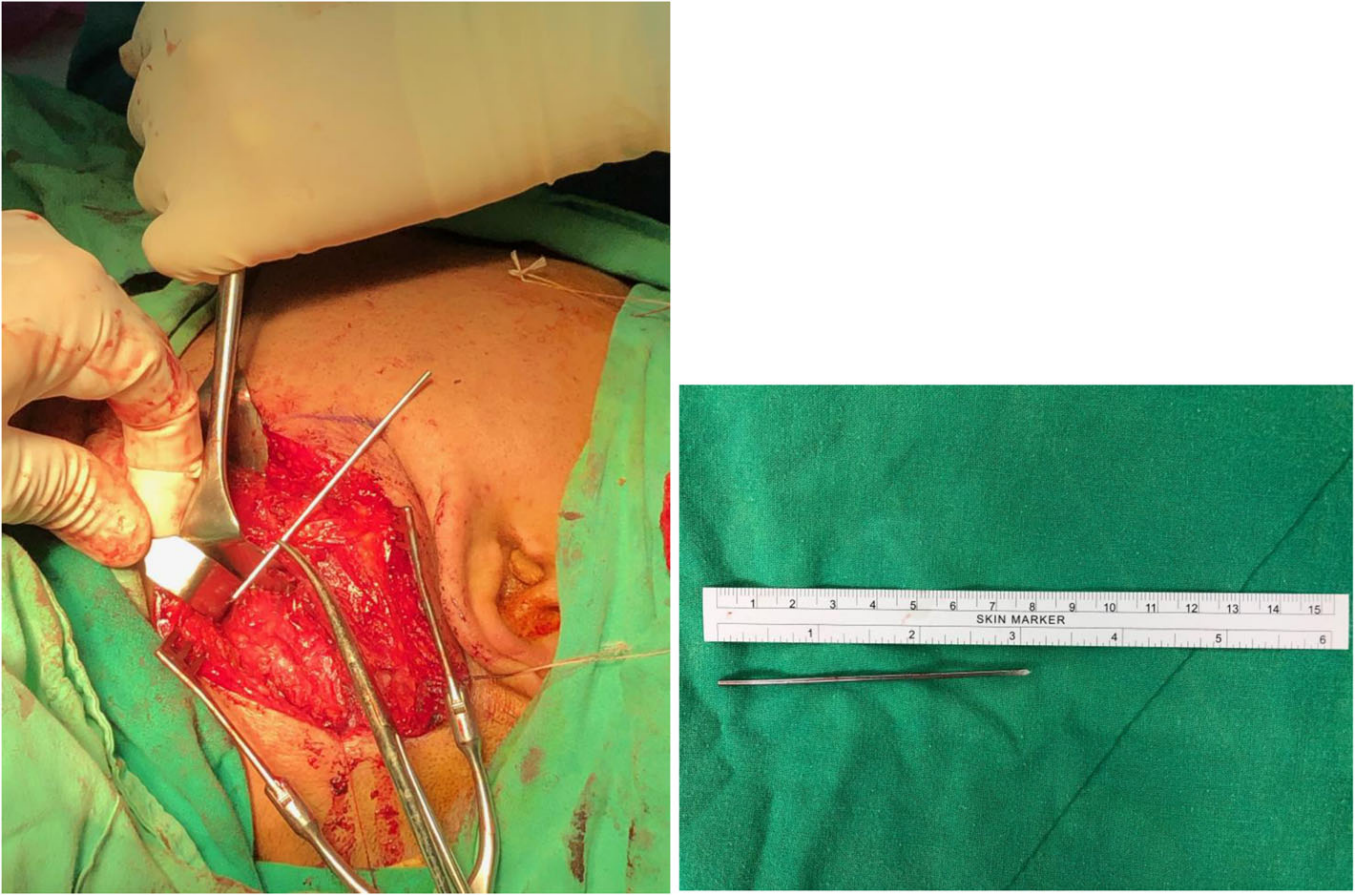
Broken Kirschner wire was found beneath left side of carotid sheath

A chest radiograph on November 16, 2018, revealed metallic internal fixation pins and wire over his right clavicle, and an unidentified object of metallic density at the left side of his upper neck. A further cervical spine radiograph was arranged, which revealed a foreign body of metallic density crossing over the C2–C4 level on the left side of his neck (Fig. 1) that indicated a loosened K-wire. He recalled that he had suffered from a right clavicle fracture which was operated on for wire fixation approximately 15 years ago, but had lost follow-up since then. Palpation of his neck revealed no palpable foreign body. His oral cavity was clear, and findings on nasopharyngoscopy were also normal. A series of chest radiographs from 3 years ago showed that the wire had migrated from the right AC joint to the left side of his neck (Fig. 2). Computed tomography angiography was performed to evaluate the position of the K-wire regarding the major vessels. The wire was removed by exploratory neck dissection under C-arm fluoroscopy assistance. The wire was found beneath the carotid sheath and removed by gentle traction along its axis (Fig. 3). His postoperation recovery was uneventful, and he was discharged 4 days later.

## Discussion and conclusion

The mechanisms of wire migration remain obscure. Several hypotheses have been postulated, including muscular activity, respiratory motion, gravity, and motion of upper extremity [5, 7–9]. In our case, our patient uses a large range of motions of his upper extremities in his work in express delivery. This may reasonably contribute to the long-range migration of the wire. The route of wire migrations can be categorized into three directions according to the axis between the wire and the spine: transversal, ascendant, and descendant [10]. However, the wire migration route in this case contained all three directions, which is rare. This case featured 3 years of continual chest radiography tracking the loosened K-wire, which gradually migrated from the right AC joint to left side of the neck, following a route of transversal, descendant, then ascendant directions (Fig. 2). To the best of our knowledge, this is the first documented case of contralateral K-wire migration without damage to any important structures or great vessels in the neck.

Some authors strongly advised that patients with K-wire fixations be followed up regularly every 4 weeks, and the wire be removed once therapeutic outcomes have been obtained [8, 11, 12]. If any sign of wire loosening or migration is present, immediate removal is required. However, it is sometimes difficult to precisely locate the migrated implant intraoperatively [5]. Furthermore, postural change such as head turning during the procedure may lead to further migration of implant. Hence, intraoperative C-arm fluoroscopy or ultrasound may provide real-time images to locate the migrated foreign body [13].

Wire migrations may occur after surgical treatments with or without clinical complaint. Moreover, all types of wires (smooth, threaded, or bent) have been reported to migrate [7, 14]. Therefore, use of wire in bone fixation – especially in the shoulder girdle region – requires extra caution during the follow-up. Patients with wire fixation should be instructed on the importance of follow-up, and routine radiograph tracking should be conducted every 4 weeks [7]. Once wire loosening or migration is noted, the implant should be removed immediately under intraoperative C-arm fluoroscopy or ultrasound assistance.

## Data Availability

Not applicable.

## Abbreviations

AC: Acromioclavicular
K-wire: Kirschner wire

## References

[1] Tan L, Sun DH, Yu T, Wang L, Zhu D, Li YH. Death Due to Intra-aortic Migration of Kirschner Wire From the Clavicle: A Case Report and Review of the Literature. Medicine (Baltimore). 2016;95(21):e3741. 10.1097/MD.0000000000003741 PMC4902362 27227938

[2] Regel JP, Pospiech J, Aalders TA, Ruchholtz S. Intraspinal migration of a Kirschner wire 3 months after clavicular fracture fixation. Neurosurg Rev. 2014;25(1–2):110–2. 10.1007/s101430100186 11954764

[3] Loncán LIC, Sempere DF, Ajuria JE. Brown-Sequard syndrome caused by a Kirschner wire as a complication of clavicular osteosynthesis. Spinal Cord. 1998;36(11):797–9. 10.1038/sj.sc.3100683 9848489

[4] Mellado JM, Calmet J, Garcia Forcada IL, Sauri A, Gine J. Early intrathoracic migration of Kirschner wires used for percutaneous osteosynthesis of a two-part humeral neck fracture: a case report. Emerg Radiol. 2004;11(1):49–52. 10.1007/s10140-004-0361-4 15278700

[5] Ballas R, Bonnel F. Endopelvic migration of a sternoclavicular K-wire. Case report and review of literature. Orthop Traumatol Surg Res. 2012;98(1):118–21. 10.1016/j.otsr.2011.09.015 22209044

[6] Foster GT, Chetty KG, Mahutte K, Kim JB, Sasse SA. Hemoptysis Due to Migration of a Fractured Kirschner Wire. Chest. 2001;119(4):1285–6. 10.1378/chest.119.4.1285 11296205

[7] Lyons FA, Rockwood CA Jr. Migration of pins used in operations on the shoulder. J Bone Joint Surg Am. 1990;72(8):1262–7. 2204634

[8] Lindsey RW, Gutowski WT. The migration of a broken pin following fixation of the acromioclavicular joint. A case report and review of the literature. Orthopedics. 1986;9(3):413–6. 10.3928/0147-7447-19860301-16 3515335

[9] Leppilahti J, Jalovaara P. Migration of Kirschner wires following fixation of the clavicle-a report of 2 cases. Acta Orthop Scand. 2009;70(5):517–9. 10.3109/17453679909000992 10622489

[10] N'da HA, Drogba KL, Konan LM, Haidara A, Varlet G. Spinal kirschner wire migration after surgical treatment of clavicular fracture or acromioclavicular joint dislocation: Report of a case and meta-analysis. Interdiscip Neurosurg. 2018;12:36–40.

[11] Fransen P, Bourgeois S, Rommens J. Kirschner wire migration causing spinal cord injury one year after internal fixation of a clavicle fracture. Acta Orthop Belg. 2007;73(3):390–2. 17715732

[12] Batin S, Ozan F, Gurbuz K, Uzun E, Kayali C, Altay T. Migration of a Broken Kirschner Wire after Surgical Treatment of Acromioclavicular Joint Dislocation. Case Rep Surg. 2016;2016:6804670. 10.1155/2016/6804670 PMC5183800 28058127

[13] Jung S, Lim H, Koh SH, Jung SW. Management of Foreign Object Migration and Surgical Removal with C-Arm Fluoroscopy. Arch Plast Surg. 2015;42(4):492–4. 10.5999/aps.2015.42.4.492 PMC4513062 26217574

[14] Cerruti P, Mangano T, Giovale M, Repetto I. Early asymptomatic intrathoracic migration of a threaded pin after proximal humeral osteosynthesis. Int J Shoulder Surg. 2016;10(1):41–3. 10.4103/0973-6042.174520 PMC4772416 26980989

